# Health economics and quality of life in a feasibility RCT of paediatric acute appendicitis: a protocol study

**DOI:** 10.1136/bmjpo-2018-000347

**Published:** 2018-09-21

**Authors:** Maria Chorozoglou, Isabel Reading, Simon Eaton, Natalie Hutchings, Nigel J Hall

**Affiliations:** 1 Southampton Health Technology Assessment Centre (SHTAC), Faculty of Medicine, University of Southampton, Southampton, UK; 2 Primary Care and Population Sciences, Faculty of Medicine, University of Southampton, Southampton, UK; 3 Developmental Biology & Cancer Programme, UCL Great Ormond Street Institute of Child Health, London, UK; 4 Southampton Clinical Trials Unit, Faculty of Medicine, University of Southampton, Southampton, UK; 5 Department of Paediatric Surgery and Urology, Southampton Children’s Hospital, University Hospital Southampton NHS Foundation Trust, Southampton, UK; 6 University Surgery Unit, Faculty of Medicine, University of Southampton, Southampton, UK

**Keywords:** health economics, paediatric surgery, costing

## Abstract

**Background:**

Acute appendicitis is one of the most common acute surgical emergencies in children and accounts for an annual cost of approximately £50 million to the National Health Service. Investigating alternative treatment options offers the best prospect of enhancing the quality of care for patients and potential opportunities for cost savings through better allocative efficiency. A feasibility randomised controlled trial (RCT) comparing a non-operative treatment pathway with appendicectomy for children with acute uncomplicated appendicitis is underway (CONTRACT feasibility RCT).

**Aims:**

The prime objective of this economic substudy conducted alongside the CONTRACT feasibility RCT is to better understand and assess: (1) cost data collection tools and cost drivers by identifying patients’ pathways and (2) patient quality of life by assessing alternative paediatric health-related quality of life (HRQoL) instruments. Outcomes from this study will inform a future efficacy RCT assessing the effectiveness and cost-effectiveness of non-operative treatment pathway for the treatment of acute uncomplicated appendicitis in children.

**Methods:**

The economic substudy will use individual-level data and will be conducted from the health system perspective over the study’s 6-month follow-up period. Microcosting will include health resource and service use, while potential benefits acquired will be measured using the HRQoL measures, Child Health Utility 9D (CHU-9D) and Euroqol-5 dimensions and 5 levels (EQ-5D-5L). We will assess the appropriateness of using the cost per quality-adjusted life year framework in the future RCT, as well as testing and identifying the most suitable HRQoL instrument.

**Conclusions:**

The outcomes of the investigational economic substudy will be used to inform the design of our future definitive RCT. However, the result from this economic study will also provide a detailed description and account of the issues inherent in paediatric Economic Evaluations Alongside Clinical Trials with an emphasis on costing methods of interventions taking place in secondary care settings.

**Trial registration number:**

ISRCTN1583043.

## Introduction

### Background

Acute appendicitis is one of the most common acute surgical emergencies in children. According to the National Schedule of Reference Costs/Healthcare Resource Groups (HRG) data, almost 14 000 operations are performed every year in the UK (<18 years). Appendicectomy procedure for a patient under 18 years old costs on average from £3072 to £5992 and accounts for an annual total cost of approximately £50 million to the National Health Service (NHS).^1^

For many years, appendicectomy has been considered the standard treatment for appendicitis, both in adults and in children. However, there is great current interest in the role of non-operative treatment (with antibiotics alone) of adults and children with acute appendicitis. Alongside evaluations of the clinical effectiveness of this alternative treatment option, it is equally important to explore the economic implications. A small number of studies have reported (mainly clinical) outcomes of non-operative treatment of acute appendicitis in children, but very little is published on how this translates into economic outcomes.^2–5^ Therefore, the economic impact of different treatment options on the health system (eg, NHS) if adopted remains largely unknown. We plan to conduct a definitive randomised controlled trial (RCT) comparing these alternative treatment options. Since recruitment to such an RCT will be challenging, we are first conducting a feasibility RCT in the UK.^6^ Alongside this feasibility RCT, we will conduct a health economic feasibility substudy to inform a future cost-effectiveness and cost–utility analysis (CUA) within our definitive RCT. Herein, we describe the protocol for this health economic substudy. We aim to address the following research questions: what are the cost implications of treating childhood appendicitis non-operatively as compared with surgery; how the costs of both treatment options compare with widely used NHS Reference Costs; and what could be the implications of differing cost methods in assessing the cost-effectiveness of appendicitis. The study will also assess two preference-based quality of life (QoL) questionnaires widely used in paediatric research, using as reference case, clinical outcomes identified through the feasibility study.

### Study design, participants, interventions and outcomes

The CONservative TReatment of Appendicitis in Children – randomised controlled Trial (CONTRACT) study is a feasibility RCT that aims to explore whether it is feasible and acceptable to conduct a multicentre RCT testing the effectiveness and cost-effectiveness of a non-operative treatment pathway for the treatment of acute uncomplicated appendicitis in children. The study is being conducted in three specialist NHS Paediatric Units in England and participants are children (age 4–15 years) with a clinical diagnosis of acute uncomplicated appendicitis. It has been estimated that 52–65 participants in the feasibility RCT will be adequate to test treatment pathway procedures. The health technology under assessment involves treatment with antibiotics (intravenous followed by oral) and regular clinical review to determine disease resolution without appendicectomy or appendicectomy in those whom the disease worsens or fails to resolve. The broad inclusion criteria reflect current clinical practice and enable the generalisability of our results for routine inpatient care. The principal outcome of the feasibility study will be recruitment rate. A full protocol of the CONTRACT feasibility RCT is published elsewhere.^6^

### Scope of the economic substudy protocol

The economic substudy will provide evidence and guidance for determining data collection tools measuring cost and benefit outcomes for our future RCT assessing the cost-effectiveness of the non-operative treatment of appendicitis. Data management will be performed by the Southampton Clinical Trials Unit, and anonymised data will be delivered for the health economic analysis. This protocol describes methods for incorporating economic evidence into an early stage of the study and has been conceptually divided into two parts: (1) measuring resource utilisation and conducting microcosting, and (2) measuring QoL and assessment of health-related QoL (HRQoL) instruments.

## Methods and analysis

### Part I: resource utilisation and costs

Costs are an important part of any economic evaluation but is a term that has different meaning across different disciplines. In Health Economics, costs are related to opportunity costs, and the question of interest is the choice between two alternatives; in other words, there are always forgone opportunities when choosing to invest in a new medical technology or health service rather than in a current treatment.^7 8^

#### Principles of costing within CONTRACT

The quality of the economic evaluation depends on the quality of the measurement of costs and outcomes.^8–10^ There are two main approaches used to measure healthcare costs: the ‘macrocosting/top-down’ and the ‘microcosting/bottom-up’ approach.^8 11–14^ The gross or macrocosting method is commonly used in cost analysis providing an overview of the effect of costs, but it has been argued that it is not appropriate in many cases for economic evaluation, because it provides limited accuracy and detail.^8 14^ In the UK, it is common practice to use the NHS Reference Costs/HRGs, which provides national tariffs as the unit costs for different services and procedures. These unit costs are calculated using the mean costs among patients and hospitals. However, these national tariffs might not represent good estimates of real costs especially for new interventions, and some reports show cost estimate discrepancies between macrocosting and microcosting, ranging between 9% and 66%.^15^ Advocates of the macrocosting approach highlight the advantages of this method, namely generalisability, easy to use and less time-consuming. Microcosting reflects the individual patient costs by identifying and collecting the actual individual resources used and estimating the economic costs of resources used. However, despite the micro-costing approach being regarded as more accurate and transparent, it is considered time-consuming and not easily applied in some settings.^14^ A general rule recommended by Beecham and Knapp^16^ is that the broader and most accurate approach is collecting individual data through a microcosting method or adopting a ‘reduced list costing’,^17^ which implies the selection of a limited number of services considered to be of most significance.

Detailed recommended methodology on costing is still lacking, but several guidelines have emerged^13 18–21^ such as a series of task force reports on methodological issues in costing methods by the International Society for Pharmacoeconomics and Outcomes Research.^20 22^ However, guidelines still vary in terms of recommended methods, and variations of costing methods affect the validity and comparability of economic evaluation results.^23 24^ Our approach in this economic study is to compare the two methods in an attempt to minimise costing bias and improve choice of instruments that will be used in our future RCT.

#### Aims

- To develop and assess resource use data collection tools in support of the future economic evaluation within our definitive future RCT.
- To conduct microcosting of both treatment pathways and to explore what are the determinants of variation in costs across settings and methods (macrocosting vs microcosting).
- To provide an economic rationale for the use of the most appropriate resource use identification, valuation and data collection tools.

#### Identifying what costs are to be included

A microcosting approach to data identification and collection will be adopted. This approach will allow identification of resource use, meaning it will be focused and able to provide rich data about the resources used in relation to managing paediatric acute appendicitis in secondary care. Each stage of the data collection refers to event pathways for activity costing so that context and information is not lost in the final outcome of each activity. The process will include identification of services, how the service works and which components of costs are incurred on delivery of each service. We will design and map processes involved in service delivery in order to identify all relevant resource use. Therefore, the details of the inpatient resource use will be collected through the design and implementation of case report forms that will be informed from hospital records (from which medical history and previous and concurrent medication will be summarised), clinical and office charts, laboratory and pharmacy records, diaries, microfiches, radiographs and correspondence. Detailed analysis of patients’ hospital records will be undertaken for the first 10 patients recruited into CONTRACT across all three participating sites. This will enable an initial inclusive list of resource use items to be created and updated based on actual patient data using microcosting principles. This work will inform the resource use data collection tool that will be used to collect data for the remainder of the recruited patients. Additionally, patient diary cards will be used to record resource use during the 14 days immediately following discharge from hospital. These will be used to collect data on use of antibiotics, pain medications and anti-inflammatory or other relevant medications, as well as productivity loss and absence from school information. Finally, a modified version of the Client Service Receipt Inventory (CSRI)^16 25^ questionnaire will be used to collect other resource use data. The CSRI is a research instrument developed in the mid-1980s to collect information on service utilisation, income, accommodation and other cost-related variables. This will include healthcare appointments and additional family-borne costs, as reported by parents of participants at 6 weeks following discharge and at 6 months.

#### Measuring and valuing resource use

Following identification of the patient’s pathway and the services used, we will design a comprehensive list of resource use items that will be included in our resource inventory collection tool. This approach will form a comprehensive health profile of service utilisation that will form the outcome of this study and will lead to identification of the main cost drivers that need to be collected in our future definitive RCT. In this part of the study, we will use a mixed method approach for the valuation of the resources used. This valuation will use unit costs from both the Personal Social Services Research Unit and the NHS Reference Costs data. Additionally, as part of our microcosting approach, we will collect and compare unit cost data from participating hospitals.^14^

#### Data collection and analysis

After choosing the items of resource use to be included in this study, we will classify them in different components depending on the characteristics of the care pathway and service systems involved. Classifying resource use and costs implies focusing on variation at individual and aggregate level by trial arm. The economic substudy at this stage will allow us to verify the relevance of this variation.

Both datasets of resource use and costs, at individual and aggregate level, will allow us to identify the main factors that influence the cost of the intervention and will form the basis for considering the main cost drivers and methodology for inclusion in our definitive RCT. We will assess data quality and missing data identifying the most appropriate approach collecting economic data alongside randomised clinical studies. Descriptive statistics will be performed to summarise data and problems identified will be discussed and presented in a relevant publication. External validation will be achieved by comparing the outcomes from our bottom-up microcosting to the NHS Reference Costs, and the HRG tariff to identify the most appropriate costing method. We envisage that in case of significant variation in the costing methods, we will be able to adopt both methods in our future cost-effectiveness analysis (CEA) in the form of sensitivity analysis. Given the importance of costs in any CEA, this proposed work will allow defining uncertainty around the CEA results and will provide an evidence base for future research.

### Part II: preference-based HRQoL instruments and the quality-adjusted life year (QALY) framework

The most commonly cited and used paediatric preference-based generic HRQoL instruments are the Health Utility Index (HUI),^26^ Euroqol 5 dimensions youth version (EQ-5D-Y)^27^ and CHU-9D.^28^ In the UK, there is a tendency towards the use of the EQ-5D-Y due to recommendation by NICE for adult population (EQ-5D-3L^29 30^). The EQ-5D-Y comprises the same 3 L as the adult version with improved wording for children despite not having a child specific value set. Euroqol states that ‘*Recent research has indicated that regular EQ-5D-3L value sets cannot be used for children and adolescents. The main reason is that health states are valued differently when described for an adult or a child*.’.

More recently, a relatively new paediatric instrument, CHU-9D, has become more widely used in the UK. This is the only preference-based HRQoL measure specifically designed and developed with children using UK general population value sets. The HUI, although a paediatric instrument, was initially developed for patients with cancer and is not used as much in the UK. We believe this is due to two reasons: first, it relies on preference values obtained from the Canadian general population and not a UK population, which might introduce some differences. Second, there is a cost attached to the use of HUI, and this could be an issue for consideration when research studies need to operate under reasonably limited budget.

#### Principles of HRQoL assessment within CONTRACT

To enable detection of any effect of our intervention on HRQoL, we will collect data using two preference-based quality of life measures. The proposed measures are: (1) the EQ-5D-5L,^31^ which comprises the same five dimensions as the EQ-5D-3L and EQ-5D-Y but five levels of severity, which is considered to significantly increase reliability and sensitivity (discriminatory power)^31 32^ and (2) the CHU-9D, the paediatric generic quality of life measure specifically designed for use in studies with children, which comprises nine dimensions.^28 33–36^ Both measures will be obtained from parent/carer proxy responses and children if 7 years or older.

#### Aims

1. To compare two alternative preference-based generic HRQoL measures commonly used in paediatric studies.
2. To identify the most appropriate HRQoL instrument for economic evaluations alongside clinical studies for children with acute uncomplicated appendicitis in tertiary care settings.
3. To assess the variation and impact of time of data collection on utility values and the QALY framework when used in this population.

#### Data collection and analysis

We will collect both HRQoL measures at baseline, discharge, 2 weeks (to determine any short-term difference in QoL that may not be apparent at later follow-up), 6 weeks, 3 months and 6 months to define the most appropriate timing of assessment in relation to other health outcomes. Evidence from this work will support the decision for the most appropriate HRQoL instrument to be used but also will provide valuable information adopting and reporting results in our future CUA in terms of cost per QALY gained. Any imbalances detected will inform sensitivity analyses and, therefore, will enrich the results from the future definitive trial. We will also assess the appropriateness of using the QALY framework in this population, in terms of identifying aspects that are excluded from the conventional QALY framework, and aspects that the QALY framework could be sensitive in regards to timing of data collection.

## Concluding remarks

Costs of different interventions are an important part of any economic evaluation to determine whether a particular intervention is better placed, in terms of the outcomes it generates, in comparison with standard care.^7 8^ The two most commonly used methods of collecting cost data are either ‘macro/top-down’ or ‘micro/bottom-up’ costing. The macrocosting uses the total budget to produce average costs per patient. This method is quicker but assumes that all patients have the same diagnosis, severity and treatment. Microcosting measures resource use by individual patient and therefore is considered more accurate detecting cost variability among patients. This method produces better quality costs but can be time-consuming and expensive.^14^ In this study, we will assess two HRQoL measures and the implications of adopting the QALY framework in our future economic evaluation. Incorporating the outcomes from this economic substudy into the feasibility stage of our RCT, and the microcosting method we adopt in doing so, we believe it will enhance our results and their applicability for healthcare decision making and for future economic evaluations.

## Supplementary Material

Reviewer comments

Author's manuscript
