# Supplementary material for: Health economics and quality of life in a feasibility RCT of paediatric acute appendicitis: a protocol study
Source: BMJ Paediatr Open. 2018 Sep 21;2(1):e000347. doi: 10.1136/bmjpo-2018-000347 (PMC6157565; doi:10.1136/bmjpo-2018-000347)
Supplement: Author's manuscript [file bmjpo-2018-000347.draft_revisions.pdf]

BMJ Paediatrics Open

BMJ Paediatrics Open is committed to open peer review. As part of this commitment we make the peer review history of every article we publish publicly available.

When an article is published we post the peer reviewers' comments and the authors' responses online. We also post the versions of the paper that were used during peer review. These are the versions that the peer review comments apply to.

The versions of the paper that follow are the versions that were submitted during the peer review process. They are not the versions of record or the final published versions. They should not be cited or distributed as the published version of this manuscript.

BMJ Paediatrics Open is an open access journal and the full, final, typeset and author-corrected version of record of the manuscript is available on our site with no access controls, subscription charges or pay-per-view fees (http://bmjpaedsopen.bmj.com).

If you have any questions on BMJ Paediatrics Open's open peer review process please email info.bmjpo@bmj.com

BMJ Paediatrics Open

HEALTH ECONOMICS PROTOCOL: MICRO-COSTING AND HRQoL ASSESSMENT IN A FEASIBILITY RCT OF PAEDIATRIC ACUTE APPENDICITIS

Journal:	*BMJ Paediatrics Open*
Manuscript ID	bmjpo-2018-000347
Article Type:	Protocol
Date Submitted by the Author:	17-Jul-2018

Complete List of Authors:	Chorozoglou, Maria; University of Southampton, Faculty of Medicine<br>Reading, Isabel ; University of Southampton , Primary Care and Population Sciences, Faculty of Medicine<br>Eaton, Simon; UCL Institute of Child Health,<br>Hutchings , Natalie; University of Southampton, Southampton Clinical Trials Unit, Faculty of Medicine<br>Hall, Nigel; University Surgery Unit

Keywords:	Health Economics, Paediatric Surgery, Costing

SCHOLARONE™
Manuscripts

Confidential: For Review Only

HEALTH ECONOMICS PROTOCOL: MICRO-COSTING AND HRQoL ASSESSMENT IN A FEASIBILITY RCT OF PAEDIATRIC ACUTE APPENDICITIS

Authors:

Maria Chorozoglou (M.Chorozoglou@soton.ac.uk)[1]; Isabel Reading (i.c.reading@soton.ac.uk)[2]; Simon Eaton (s.eaton@ucl.ac.uk)[3]; Natalie Hutchings (n.j.hutchings@soton.ac.uk)[4]; and Nigel J Hall (n.j.hall@soton.ac.uk)[5],[6]

Author for correspondence:

Maria Chorozoglou,
Senior Research Fellow - Health Economist
Southampton Health Technology Assessment Centre,
Faculty of Medicine, University of Southampton
The University of Southampton Science Park
Alpha House, Enterprise Road, Southampton SO17 1BJ, UK
E-mail: M.Chorozoglou@soton.ac.uk

[1] Southampton Health Technology Assessment Centre (SHTAC), Faculty of Medicine, University of Southampton,
[2] Primary Care and Population Sciences, Faculty of Medicine, University of Southampton
[3] UCL Great Ormond Street Institute of Child Health
[4] Southampton Clinical Trials Unit, Faculty of Medicine, University of Southampton
[5] Department of Paediatric Surgery and Urology, Southampton Children's Hospital, University Hospital Southampton NHS Foundation Trust, UK
[6] University Surgery Unit, Faculty of Medicine, University of Southampton

Confidential: For Review Only

What is known about the subject – followed by a maximum of 3 brief statements (no more than 25 words per statement)

1. Acute appendicitis is one of the most common acute surgical emergencies in children and there is great current interest in the role of non-operative treatment.
2. The economic impact of different treatment options on the health system (e.g. NHS) if adopted remains largely unknown.
3. The quality of any economic evaluation depends on the quality of the measurement of costs and outcomes, despite this very few HE protocols are published.

What this study hopes to add – followed by a maximum of 3 brief statements (no more than 25 words per statement)

1. This study protocol provides a comprehensive account of the work that will be carried out offering exposure and dissemination of research activity.
2. Presents and describes the research questions that will be answered through this study and by doing so increases transparency.
3. Adds to evidence base in paediatric surgical research in terms of methodology for economic studies.

- **Authors' contributions**: state how each author was involved in writing the protocol

The lead health economist (MC) designed the Health Economic Analysis Plan and prepared this manuscript; the lead statistician (IR) contributed to the design of the study; the principal investigator (NH) is involved in all aspects of the study; All co-authors contributed to the preparation and approval of this manuscript.

- **Funding statement**: preferably worded in one of two ways:

The authors acknowledge funding received from the UK National Institute for Health Research (NIHR) Health Technology Assessment (HTA) Board (14/192/90)

- **Competing interests statement**

No competing interest has been declared from any of the authors.

Confidential: For Review Only

ABSTRACT

Background: Acute appendicitis is one of the most common acute surgical emergencies in children and accounts for an annual cost of approximately £50 million to the NHS. Investigating alternative treatment options offers not only the best prospect of enhancing the quality of care for patients but also potential opportunities for cost savings through better allocative efficiency. A feasibility Randomised Controlled Trial (RCT) comparing a non-operative treatment pathway with appendicectomy for children with acute uncomplicated appendicitis is underway (CONTRACT feasibility RCT).

Aims: The prime objective of this economic sub-study conducted alongside the CONTRACT feasibility RCT is to better understand and assess: (i) cost data collection tools and cost drivers by identifying patients' pathways and (ii) patient Quality of Life (QoL) by assessing alternative paediatric Health Related Quality of Life (HRQoL) instruments. Outcomes from this study will inform a future efficacy RCT assessing the effectiveness and cost-effectiveness of non-operative treatment pathway for the treatment of acute uncomplicated appendicitis in children.

Methods: The economic sub-study will use individual-level data and will be conducted from the health system perspective over the study's 6-month follow-up period. Micro-costing will include health resource and service use, while potential benefits acquired will be measured using the HRQoL measures, CHU-9D and EQ-5D-5L. We will assess the appropriateness of using the cost per QALY framework in the future RCT, as well as testing and identifying the most suitable HRQoL instrument.

Conclusions: The outcomes of the investigational economic sub-study will be used to inform the design of our future definitive RCT. However, the result from this economic study will also provide a detailed description and account of the issues inherent in paediatric Economic Evaluations Alongside Clinical Trials (EEACT) with an emphasis on costing methods of interventions taking place in secondary care settings.

Word count:
Abstract 292
Main manuscript 2587

Confidential: For Review Only

INTRODUCTION

Background

Acute appendicitis is one of the most common acute surgical emergencies in children. According to the National Schedule of Reference Costs/HRG (Healthcare Resource Groups) data, almost fourteen thousand operations are performed every year in the UK (<18y). Appendicectomy procedure for a patient under 18 years old, costs on average from £3,072 to £5,992 and accounts for an annual total cost of approximately £50 million to the NHS (1).

For many years, appendicectomy has been considered the standard treatment for appendicitis, both in adults and in children. However, there is great current interest in the role of non-operative treatment (with antibiotics alone) of adults and children with acute appendicitis. Alongside evaluations of the clinical effectiveness of this alternative treatment option, it is equally important to explore the economic implications. A small number of studies have reported (mainly clinical) outcomes of non-operative treatment of acute appendicitis in children, but very little is published on how this translates into economic outcomes (2-5). Therefore, the economic impact of different treatment options on the health system (e.g. NHS) if adopted remains largely unknown. We plan to conduct a definitive RCT comparing these alternative treatment options. Since recruitment to such a RCT will be challenging, we are first conducting a feasibility RCT in the UK (6). Alongside this feasibility RCT, we will conduct a health economic feasibility sub-study to inform a future cost-effectiveness and cost-utility analysis within our definitive RCT. Herein we describe the protocol for this health economic sub-study. We aim to address the following research questions: what are the cost implications of treating childhood appendicitis non-operatively as compared to surgery; how the costs of both treatment options compare to widely used NHS Reference Costs; and what could be the implications of differing cost methods in assessing the cost-effectiveness of appendicitis. The study will also assess two preference-based QoL questionnaires widely used in paediatric research, using as reference case, clinical outcomes identified through the feasibility study.

Study design, participants, interventions, outcomes

Confidential: For Review Only

The CONservative TReatment of Appendicitis in Children – randomised controlled Trial (CONTRACT) study is a feasibility RCT that aims to explore whether it is feasible and acceptable to conduct a multi-centre randomised controlled trial testing the effectiveness and cost-effectiveness of a non-operative treatment pathway for the treatment of acute uncomplicated appendicitis in children. The study is being conducted in three specialist NHS Paediatric Units in England and participants are children (age 4-15 years) with a clinical diagnosis of acute uncomplicated appendicitis. It has been estimated that 52-65 participants in the feasibility RCT will be adequate to test treatment pathway procedures. The health technology under assessment involves treatment with antibiotics (intravenous followed by oral) and regular clinical review to determine disease resolution without appendicectomy, or appendicectomy in those whom the disease worsens or fails to resolve. The broad inclusion criteria reflect current clinical practice and enable the generalisability of our results for routine in-patient care. The principal outcome of the feasibility study will be recruitment rate. A full protocol of the CONTRACT feasibility RCT is published elsewhere (6). The study, including the economic sub-study, has been approved by the South Central – Hampshire A Research Ethics Committee (16/SC/0596). The feasibility RCT was registered on 8th February 2017 (http://www.isrctn.com/ISRCTN15830435).

Scope of the economic sub-study protocol

The economic sub-study will provide evidence and guidance for determining data collection tools measuring cost and benefit outcomes for our future RCT assessing the cost-effectiveness of the non-operative treatment of appendicitis. Data management will be performed by the Southampton Clinical Trials Unit (SCTU) and anonymised data will be delivered for the health economic analysis. This protocol describes methods for incorporating economic evidence into an early stage of the study and has been conceptually divided into two parts: (i) measuring resource utilisation and conducting micro-costing, and (ii) measuring QoL and assessment of HRQoL instruments.

METHODS AND ANALYSIS

PART I: RESOURCE UTILISATION & COSTS

Confidential: For Review Only

Costs are an important part of any economic evaluation but is a term that has different meaning across different disciplines. In Health Economics, costs are related to opportunity costs and the question of interest is the choice between two alternatives, in other words there are always forgone opportunities when choosing to invest in a new medical technology or health service rather than in a current treatment (7, 8).

Principles of costing within CONTRACT

The quality of the economic evaluation depends on the quality of the measurement of costs and outcomes (8-10). There are two main approaches used to measure health care costs: the “macro-costing/top-down” and the “micro-costing/bottom-up” approach (8, 11-14). The gross or macro-costing method is commonly used in cost analysis providing an overview of the effect of costs, but it has been argued that it is not appropriate in many cases for economic evaluation because it provides limited accuracy and detail (8, 14). In the UK, it is common practice to use the NHS Reference Costs/HRGs which provides National tariffs as the unit costs for different services and procedures. These unit costs are calculated using the mean costs among patients and hospitals. However, these national tariffs might not represent good estimates of real costs especially for new interventions and some reports show cost estimate discrepancies between macro-costing and micro-costing, ranging between 9% and 66% (15). Advocates of the macro-costing approach highlight the advantages of this method, namely generalisability, easy to use and less time consuming. Micro-costing reflects the individual patient costs by identifying and collecting the actual individual resources used and estimating the economic costs of resources utilised. However, despite the micro-costing approach being regarded as more accurate and transparent, it is considered time consuming and not easily applied in some settings (14). A general rule recommended by Beecham and Knapp (16) is that the broader and most accurate approach is collecting individual data through a micro-costing method or adopting a “reduced list costing” (17) which implies the selection of a limited number of services considered to be of most significance.

Detailed recommended methodology on costing is still lacking but several guidelines have emerged (13, 18-21) such as a series of task force reports on methodological issues in costing methods by the International Society for Pharmacoeconomics and

Confidential: For Review Only

Outcomes Research (20, 22). However, guidelines still vary in terms of recommended methods and variations of costing methods affect the validity and comparability of economic evaluation results (23, 24). Our approach in this economic study is to compare the two methods in an attempt to minimise costing bias and improve choice of instruments that will be used in our future RCT.

Aims:

- To develop and assess resource use data collection tools in support of the future economic evaluation within our definitive future RCT.
- To conduct micro-costing of both treatment pathways and to explore what are the determinants of variation in costs across settings and methods (macro- vs. micro-costing).
- To provide an economic rationale for the use of the most appropriate resource use identification, valuation and data collection tools.

Identifying what costs are to be included

A micro-costing approach to data identification and collection will be adopted. This approach will allow identification of resource use, meaning it will be focused and able to provide rich data about the resources used in relation to managing paediatric acute appendicitis in secondary care. Each stage of the data collection refers to event pathways for activity costing so that context and information is not lost in the final outcome of each activity. The process will include identification of services, how the service works and which components of costs are incurred upon delivery of each service. We will design and map processes involved in service delivery in order to identify all relevant resource use. Therefore, the details of the in-patient resource use will be collected through the design and implementation of Case Report Forms (CRFs) that will be informed from hospital records (from which medical history and previous and concurrent medication will be summarised), clinical and office charts, laboratory and pharmacy records, diaries, microfiches, radiographs, and correspondence. Detailed analysis of patients' hospital records will be undertaken for the first 10 patients recruited into CONTRACT across all three participating sites. This will enable an initial inclusive list of resource use items to be created and updated based on actual patient data using micro-costing principles. This work will inform the resource use data collection tool that will be used to collect data for the remainder of the recruited patients. Additionally, patient diary cards will be used to

Confidential: For Review Only

record resource use during the 14 days immediately following discharge from hospital. These will be used to collect data on use of antibiotics, pain medications and anti-inflammatory, or other relevant medications, as well as productivity loss and absence from school information. Finally, a modified version of the Client Service Receipt Inventory (CSRI) (16, 25) questionnaire will be used to collect other resource use data. The CSRI is a research instrument developed in the mid 1980s to collect information on service utilisation, income, accommodation and other cost-related variables. This will include health care appointments and additional family borne costs, as reported by parents of participants at 6-weeks following discharge and at 6-months.

Measuring and valuing resource use

Following identification of the patient's pathway and the services utilised, we will design a comprehensive list of resource use items that will be included in our resource inventory collection tool. This approach will not only form a comprehensive health profile of service utilisation that will form the outcome of this study, but will also lead to identification of the main cost drivers that need to be collected in our future definitive RCT. In this part of the study, we will use a mixed method approach for the valuation of the resources used. This valuation will utilise unit costs from both the PSSRU (Personal Social Services Research Unit) and the NHS Reference Costs data. Additionally, as part of our micro-costing approach we will collect and compare unit cost data from participating hospitals (14).

Data collection and analysis

After choosing the items of resource use to be included in this study, we will classify them in different components depending on the characteristics of the care pathway and service systems involved. Classifying resource use and costs implies focusing on variation at individual and aggregate level by trial arm. The economic sub-study at this stage will allow us to verify the relevance of this variation.

Both datasets of resource use and costs, at individual and aggregate level, will allow us to identify the main factors that influence the cost of the intervention, and will form the basis for considering the main cost drivers and methodology for inclusion in our definitive RCT. We will assess data quality and missing data identifying the most appropriate approach collecting economic data alongside randomised clinical

Confidential: For Review Only

studies. Descriptive statistics will be performed to summarise data and problems identified will be discussed and presented in a relevant publication. External validation will be achieved by comparing the outcomes from our bottom-up micro-costing to the NHS Reference Cost and the HRG tariff to identify the most appropriate costing method. We envisage that in case of significant variation in the costing methods we will be able to adopt both methods in our future cost-effectiveness analysis (CEA) in the form of sensitivity analysis. Given the importance of costs in any CEA, this proposed work will allow defining uncertainty around the CEA results and will provide an evidence base for future research.

PART II: PREFERENCE-BASED HRQoL INSTRUMENTS & THE QALY FRAMEWORK

The most commonly cited and used paediatric preference-based generic HRQoL instruments are the HUI (26), EQ-5D-Y (27) and CHU-9D (28). In the UK, there is a tendency towards the use of the EQ-5D-Y due to recommendation by NICE for adult population (EQ-5D-3L (29, 30)). The EQ-5D-Y comprises the same 3L as the adult version with improved wording for children despite not having a child specific value set. Euroqol states that *"Recent research has indicated that regular EQ-5D-3L value sets cannot be used for children and adolescents. The main reason is that health states are valued differently when described for an adult or a child."*

More recently a relatively new paediatric instrument, CHU-9D, has become more widely used in the UK. This is the only preference-based HRQoL measure specifically designed and developed with children using UK general population value sets. The HUI, although a paediatric instrument, was initially developed for patients with cancer and is not used as much in the UK. We believe this is due to two reasons: firstly, it relies on preference values obtained from the Canadian general population and not a UK population which might introduce some differences. Secondly, there is a cost attached to the use of HUI and this could be an issue for consideration when research studies need to operate under reasonably limited budget.

Principles of HRQoL assessment within CONTRACT

Confidential: For Review Only

To enable detection of any effect of our intervention on HRQoL we will collect data using two preference-based quality of life measures. The proposed measures are: (i) the EQ-5D-5L (31) which comprises the same 5 dimensions as the EQ-5D-3L and EQ-5D-Y but 5 levels of severity, which is considered to significantly increase reliability and sensitivity (discriminatory power) (31, 32) and (ii) the CHU-9D, the paediatric generic quality of life measure specifically designed for use in studies with children, which comprises 9 dimensions (28, 33-36). Both measures will be obtained from parent/carer proxy responses and children if 7-year or older.

Aims:

1. To compare two alternative preference-based generic HRQoL measures commonly used in paediatric studies.
2. To identify the most appropriate HRQoL instrument for economic evaluations alongside clinical studies for children with acute uncomplicated appendicitis in tertiary care settings.
3. To assess the variation and impact of time of data collection on utility values and the QALY framework when used in this population.

Data collection and analysis

We will collect both HRQoL measures at baseline, discharge, 2 weeks (to determine any short-term difference in QoL that may not be apparent at later follow-up), 6 weeks, 3 months, and 6 months, to define the most appropriate timing of assessment in relation to other health outcomes. Evidence from this work will support the decision for the most appropriate HRQoL instrument to be used, but also will provide valuable information adopting and reporting results in our future cost-utility analysis (CUA) in terms of cost per QALY gained. Any imbalances detected will inform sensitivity analyses and therefore, will enrich the results from the future definitive trial. We will also assess the appropriateness of using the QALY framework in this population, in terms of identifying aspects that are excluded from the conventional QALY framework, and aspects that the QALY framework could be sensitive in regards to timing of data collection.

CONCLUDING REMARKS

Costs of different interventions are an important part of any economic evaluation to determine whether a particular intervention is better placed, in terms of the outcomes

Confidential: For Review Only

it generates, in comparison to standard care (7, 8). The two most commonly used methods of collecting cost data are either “macro/top-down” or “micro/bottom-up” costing. The macro-costing uses the total budget to produce average costs per patient. This method is quicker but assumes that all patients have the same diagnosis, severity and treatment. Micro-costing measures resource use by individual patient, and therefore is considered more accurate detecting cost variability among patients. This method produces better quality costs but can be time-consuming and expensive (14). In this study we will assess two HRQoL measures and the implications of adopting the QALY framework in our future economic evaluation. Incorporating the outcomes from this economic sub-study into the feasibility stage of our RCT, and the micro-costing method we adopt in doing so, we believe it will enhance our results and their applicability for healthcare decision-making and for future economic evaluations.

Confidential: For Review Only

References:

1. Department of Health. NHS Reference Costs 2016-17. In: https://improvement.nhs.uk/resources/reference-costs/ LaM-J, editor. 2017.
2. Armstrong J, Merritt N, Jones S, Scott L, Bütter A. Non-operative management of early, acute appendicitis in children: Is it safe and effective? Journal of Pediatric Surgery. 2014;49(5):782-5.
3. Hartwich J, Luks FI, Watson-Smith D, Kurkchubasche AG, Muratore CS, Wills HE, et al. Nonoperative treatment of acute appendicitis in children: A feasibility study. J Pediatr Surg. 2016;51(1):111-6.
4. Minneci PC, Sulkowski JP, Nacion KM, Mahida JB, Cooper JN, Moss RL, et al. Feasibility of a nonoperative management strategy for uncomplicated acute appendicitis in children. J Am Coll Surg. 2014;219(2):272-9.
5. Svensson JF, Patkova B, Almstrom M, Naji H, Hall NJ, Eaton S, et al. Nonoperative treatment with antibiotics versus surgery for acute nonperforated appendicitis in children: a pilot randomized controlled trial. Ann Surg. 2015;261(1):67-71.
6. Hutchings N, Wood W, Reading I, Walker E, Blazeby JM, Van't Hoff W, et al. CONTRACT Study - CONservative TReatment of Appendicitis in Children (feasibility): study protocol for a randomised controlled Trial. Trials. 2018;19(1):153.
7. Drummond M MA. Economic evaluation in health care: merging theory with practice. 2nd ed. New York: Oxford University Press; 2004.
8. Drummond M, Sculpher M, Claxton K, Stoddart G, Torrance G. Methods for the economic evaluation of health care programmes. NY: Oxford University Press; 2015.
9. Luce BR, Elixhauser A. Estimating costs in the economic evaluation of medical technologies. Int J Technol Assess Health Care. 1990;6(1):57-75.
10. Riewpaiboon A, Malaroje S, Kongsawatt S. Effect of costing methods on unit cost of hospital medical services. Trop Med Int Health. 2007;12(4):554-63.
11. Smith MW, Barnett PG. Direct measurement of health care costs. Med Care Res Rev. 2003;60(3 Suppl):74s-91s.
12. Slothuus U. An Evaluation of Selected Literature on the Measurement of Costs in Health Economics. 2000.
13. Mogyorosy ZaS, P. The main methodological issues in costing health care services: A literature review. 2005.
14. Byford SM, D. Sefton, T. Because it's worth it: a practical guide to conducting economic evaluations in the social welfare field. London: York Publishing Services Ltd; 2003.
15. Heerey A, McGowan B, Ryan M, Barry M. Microcosting versus DRGs in the provision of cost estimates for use in pharmacoeconomic evaluation. Expert Rev Pharmacoecon Outcomes Res. 2002;2(1):29-33.
16. Beecham JaK, M. Costing psychiatric interventions. G. T, editor. London2001.
17. Knapp M, Beecham J. Reduced list costings: examination of an informed short cut in mental health research. Health Econ. 1993;2(4):313-22.
18. Garrison LP, Jr., Mansley EC, Abbott TA, 3rd, Bresnahan BW, Hay JW, Smeeding J. Good research practices for measuring drug costs in cost-effectiveness analyses: a societal perspective: the ISPOR Drug Cost Task Force report--Part II. Value Health. 2010;13(1):8-13.
19. Shearer J, McCrone P, Romeo R. Economic Evaluation of Mental Health Interventions: A Guide to Costing Approaches. Pharmacoeconomics. 2016;34(7):651-64.

Confidential: For Review Only

20. Oostenbrink JB, Koopmanschap MA, Rutten FF. Standardisation of costs: the Dutch Manual for Costing in economic evaluations. Pharmacoeconomics. 2002;20(7):443-54.
21. Barnett PG. An improved set of standards for finding cost for cost-effectiveness analysis. Med Care. 2009;47(7 Suppl 1):S82-8.
22. Husereau D, Drummond M, Petrou S, Carswell C, Moher D, Greenberg D, et al. Consolidated Health Economic Evaluation Reporting Standards (CHEERS) statement. BMJ : British Medical Journal. 2013;346.
23. Adam T, Koopmanschap MA, Evans DB. Cost-effectiveness analysis: can we reduce variability in costing methods? Int J Technol Assess Health Care. 2003;19(2):407-20.
24. Edwards RT, Charles JM, Lloyd-Williams H. Public health economics: a systematic review of guidance for the economic evaluation of public health interventions and discussion of key methodological issues. BMC Public Health. 2013;13:1001.
25. Beecham J. Collecting Information: the Client Service Receipt Interview. Mental Health Research. 1994;1,:6-8.
26. Horsman J, Furlong W, Feeny D, Torrance G. The Health Utilities Index (HUI(®)): concepts, measurement properties and applications. Health and Quality of Life Outcomes. 2003;1:54-.
27. Wille N, Badia X, Bonsel G, Burstrom K, Cavrini G, Devlin N, et al. Development of the EQ-5D-Y: a child-friendly version of the EQ-5D. Qual Life Res. 2010;19(6):875-86.
28. Stevens K. Assessing the performance of a new generic measure of health-related quality of life for children and refining it for use in health state valuation. Appl Health Econ Health Policy. 2011;9(3):157-69.
29. Dolan P. EuroQol--a new facility for the measurement of health-related quality of life. Health Policy. 1990;16(3):199-208.
30. Kind P, Dolan P, Gudex C, Williams A. Variations in population health status: results from a United Kingdom national questionnaire survey. Bmj. 1998;316(7133):736-41.
31. Herdman M, Gudex C, Lloyd A, Janssen M, Kind P, Parkin D, et al. Development and preliminary testing of the new five-level version of EQ-5D (EQ-5D-5L). Qual Life Res. 2011;20(10):1727-36.
32. Janssen MF, Pickard AS, Golicki D, Gudex C, Niewada M, Scalone L, et al. Measurement properties of the EQ-5D-5L compared to the EQ-5D-3L across eight patient groups: a multi-country study. Qual Life Res. 2013;22(7):1717-27.
33. Stevens K. Developing a descriptive system for a new preference-based measure of health-related quality of life for children. Qual Life Res. 2009;18(8):1105-13.
34. Stevens KJ, Freeman JV. An assessment of the psychometric performance of the Health Utilities Index 2 and 3 in children following discharge from a U.K. pediatric intensive care unit. Pediatr Crit Care Med. 2012;13(4):387-92.
35. Stevens K. Valuation of the Child Health Utility 9D Index. Pharmacoeconomics. 2012;30(8):729-47.
36. Stevens K, Ratcliffe J. Measuring and valuing health benefits for economic evaluation in adolescence: an assessment of the practicality and validity of the child health utility 9D in the Australian adolescent population. Value Health. 2012;15(8):1092-9.

Confidential: For Review Only

BMJ Paediatrics Open

Protocol of Health Economics and Quality of Life in a feasibility RCT of Paediatric Acute Appendicitis

Journal:	*BMJ Paediatrics Open*
Manuscript ID	bmjpo-2018-000347.R1
Article Type:	Protocol
Date Submitted by the Author:	27-Jul-2018

Complete List of Authors:	Chorozoglou, Maria; University of Southampton, Faculty of Medicine<br>Reading, Isabel ; University of Southampton , Primary Care and Population Sciences, Faculty of Medicine<br>Eaton, Simon; UCL Institute of Child Health,<br>Hutchings , Natalie; University of Southampton, Southampton Clinical Trials Unit, Faculty of Medicine<br>Hall, Nigel; University Surgery Unit

Keywords:	Health Economics, Paediatric Surgery, Costing

SCHOLARONE™
Manuscripts

Confidential: For Review Only

Protocol of Health Economics and Quality of Life in a feasibility RCT of Paediatric Acute Appendicitis

Authors:

Maria Chorozoglou (M.Chorozoglou@soton.ac.uk)[1]; Isabel Reading (i.c.reading@soton.ac.uk)[2]; Simon Eaton (s.eaton@ucl.ac.uk)[3]; Natalie Hutchings (n.j.hutchings@soton.ac.uk)[4]; and Nigel J Hall (n.j.hall@soton.ac.uk)[5],[6]

Author for correspondence:

Maria Chorozoglou,
Senior Research Fellow - Health Economist
Southampton Health Technology Assessment Centre,
Faculty of Medicine, University of Southampton
The University of Southampton Science Park
Alpha House, Enterprise Road, Southampton SO17 1BJ, UK
E-mail: M.Chorozoglou@soton.ac.uk

Confidential: For Review Only

[1] Southampton Health Technology Assessment Centre (SHTAC), Faculty of Medicine, University of Southampton,
[2] Primary Care and Population Sciences, Faculty of Medicine, University of Southampton
[3] UCL Great Ormond Street Institute of Child Health
[4] Southampton Clinical Trials Unit, Faculty of Medicine, University of Southampton
[5] Department of Paediatric Surgery and Urology, Southampton Children's Hospital, University Hospital Southampton NHS Foundation Trust, UK
[6] University Surgery Unit, Faculty of Medicine, University of Southampton

What is known about the subject – followed by a maximum of 3 brief statements (no more than 25 words per statement)

1. Acute appendicitis is one of the most common acute surgical emergencies in children and there is great current interest in the role of non-operative treatment.
2. The economic impact of different treatment options on the health system (e.g. NHS) if adopted remains largely unknown.
3. The quality of any economic evaluation depends on the quality of the measurement of costs and outcomes, despite this very few HE protocols are published.

What this study hopes to add – followed by a maximum of 3 brief statements (no more than 25 words per statement)

1. This study proposes a comprehensive methodological approach that could be adopted in an early stage of assessment of a medical technology.
2. Offers transparency enhancing feasibility results and their applicability for healthcare decision making.
3. Adds to evidence base in paediatric surgical research in terms of methodology of economic studies.

- **Authors' contributions**: state how each author was involved in writing the protocol

The lead health economist (MC) designed the Health Economic Analysis Plan and prepared this manuscript; the lead statistician (IR) contributed to the design of the study; the principal investigator (NH) is involved in all aspects of the study; All co-authors contributed to the preparation and approval of this manuscript.

- **Funding statement**: preferably worded in one of two ways:

The authors acknowledge funding received from the UK National Institute for Health Research (NIHR) Health Technology Assessment (HTA) Board (14/192/90)

- **Competing interests statement**

No competing interest has been declared from any of the authors.

Confidential: For Review Only

ABSTRACT

Background: Acute appendicitis is one of the most common acute surgical emergencies in children and accounts for an annual cost of approximately £50 million to the NHS. Investigating alternative treatment options offers not only the best prospect of enhancing the quality of care for patients but also potential opportunities for cost savings through better allocative efficiency. A feasibility Randomised Controlled Trial (RCT) comparing a non-operative treatment pathway with appendicectomy for children with acute uncomplicated appendicitis is underway (CONTRACT feasibility RCT).

Aims: The prime objective of this economic sub-study conducted alongside the CONTRACT feasibility RCT is to better understand and assess: (i) cost data collection tools and cost drivers by identifying patients' pathways and (ii) patient Quality of Life (QoL) by assessing alternative paediatric Health Related Quality of Life (HRQoL) instruments. Outcomes from this study will inform a future efficacy RCT assessing the effectiveness and cost-effectiveness of non-operative treatment pathway for the treatment of acute uncomplicated appendicitis in children.

Methods: The economic sub-study will use individual-level data and will be conducted from the health system perspective over the study's 6-month follow-up period. Micro-costing will include health resource and service use, while potential benefits acquired will be measured using the HRQoL measures, CHU-9D and EQ-5D-5L. We will assess the appropriateness of using the cost per QALY framework in the future RCT, as well as testing and identifying the most suitable HRQoL instrument.

Conclusions: The outcomes of the investigational economic sub-study will be used to inform the design of our future definitive RCT. However, the result from this economic study will also provide a detailed description and account of the issues inherent in paediatric Economic Evaluations Alongside Clinical Trials (EEACT) with an emphasis on costing methods of interventions taking place in secondary care settings.

Word count:
Abstract 292
Main manuscript 2587

Confidential: For Review Only

INTRODUCTION

Background

Acute appendicitis is one of the most common acute surgical emergencies in children. According to the National Schedule of Reference Costs/HRG (Healthcare Resource Groups) data, almost fourteen thousand operations are performed every year in the UK (<18y). Appendicectomy procedure for a patient under 18 years old, costs on average from £3,072 to £5,992 and accounts for an annual total cost of approximately £50 million to the NHS (1).

For many years, appendicectomy has been considered the standard treatment for appendicitis, both in adults and in children. However, there is great current interest in the role of non-operative treatment (with antibiotics alone) of adults and children with acute appendicitis. Alongside evaluations of the clinical effectiveness of this alternative treatment option, it is equally important to explore the economic implications. A small number of studies have reported (mainly clinical) outcomes of non-operative treatment of acute appendicitis in children, but very little is published on how this translates into economic outcomes (2-5). Therefore, the economic impact of different treatment options on the health system (e.g. NHS) if adopted remains largely unknown. We plan to conduct a definitive RCT comparing these alternative treatment options. Since recruitment to such a RCT will be challenging, we are first conducting a feasibility RCT in the UK (6). Alongside this feasibility RCT, we will conduct a health economic feasibility sub-study to inform a future cost-effectiveness and cost-utility analysis within our definitive RCT. Herein we describe the protocol for this health economic sub-study. We aim to address the following research questions: what are the cost implications of treating childhood appendicitis non-operatively as compared to surgery; how the costs of both treatment options compare to widely used NHS Reference Costs; and what could be the implications of differing cost methods in assessing the cost-effectiveness of appendicitis. The study will also assess two preference-based QoL questionnaires widely used in paediatric research, using as reference case, clinical outcomes identified through the feasibility study.

Study design, participants, interventions, outcomes

Confidential: For Review Only

The CONservative TReatment of Appendicitis in Children – randomised controlled Trial (CONTRACT) study is a feasibility RCT that aims to explore whether it is feasible and acceptable to conduct a multi-centre randomised controlled trial testing the effectiveness and cost-effectiveness of a non-operative treatment pathway for the treatment of acute uncomplicated appendicitis in children. The study is being conducted in three specialist NHS Paediatric Units in England and participants are children (age 4-15 years) with a clinical diagnosis of acute uncomplicated appendicitis. It has been estimated that 52-65 participants in the feasibility RCT will be adequate to test treatment pathway procedures. The health technology under assessment involves treatment with antibiotics (intravenous followed by oral) and regular clinical review to determine disease resolution without appendicectomy, or appendicectomy in those whom the disease worsens or fails to resolve. The broad inclusion criteria reflect current clinical practice and enable the generalisability of our results for routine in-patient care. The principal outcome of the feasibility study will be recruitment rate. A full protocol of the CONTRACT feasibility RCT is published elsewhere (6). The study, including the economic sub-study, has been approved by the South Central – Hampshire A Research Ethics Committee (16/SC/0596). The feasibility RCT was registered on 8th February 2017 (http://www.isrctn.com/ISRCTN15830435).

Scope of the economic sub-study protocol

The economic sub-study will provide evidence and guidance for determining data collection tools measuring cost and benefit outcomes for our future RCT assessing the cost-effectiveness of the non-operative treatment of appendicitis. Data management will be performed by the Southampton Clinical Trials Unit (SCTU) and anonymised data will be delivered for the health economic analysis. This protocol describes methods for incorporating economic evidence into an early stage of the study and has been conceptually divided into two parts: (i) measuring resource utilisation and conducting micro-costing, and (ii) measuring QoL and assessment of HRQoL instruments.

METHODS AND ANALYSIS

PART I: RESOURCE UTILISATION & COSTS

Confidential: For Review Only

Costs are an important part of any economic evaluation but is a term that has different meaning across different disciplines. In Health Economics, costs are related to opportunity costs and the question of interest is the choice between two alternatives, in other words there are always forgone opportunities when choosing to invest in a new medical technology or health service rather than in a current treatment (7, 8).

Principles of costing within CONTRACT

The quality of the economic evaluation depends on the quality of the measurement of costs and outcomes (8-10). There are two main approaches used to measure health care costs: the “macro-costing/top-down” and the “micro-costing/bottom-up” approach (8, 11-14). The gross or macro-costing method is commonly used in cost analysis providing an overview of the effect of costs, but it has been argued that it is not appropriate in many cases for economic evaluation because it provides limited accuracy and detail (8, 14). In the UK, it is common practice to use the NHS Reference Costs/HRGs which provides National tariffs as the unit costs for different services and procedures. These unit costs are calculated using the mean costs among patients and hospitals. However, these national tariffs might not represent good estimates of real costs especially for new interventions and some reports show cost estimate discrepancies between macro-costing and micro-costing, ranging between 9% and 66% (15). Advocates of the macro-costing approach highlight the advantages of this method, namely generalisability, easy to use and less time consuming. Micro-costing reflects the individual patient costs by identifying and collecting the actual individual resources used and estimating the economic costs of resources utilised. However, despite the micro-costing approach being regarded as more accurate and transparent, it is considered time consuming and not easily applied in some settings (14). A general rule recommended by Beecham and Knapp (16) is that the broader and most accurate approach is collecting individual data through a micro-costing method or adopting a “reduced list costing” (17) which implies the selection of a limited number of services considered to be of most significance.

Detailed recommended methodology on costing is still lacking but several guidelines have emerged (13, 18-21) such as a series of task force reports on methodological issues in costing methods by the International Society for Pharmacoeconomics and

Confidential: For Review Only

Outcomes Research (20, 22). However, guidelines still vary in terms of recommended methods and variations of costing methods affect the validity and comparability of economic evaluation results (23, 24). Our approach in this economic study is to compare the two methods in an attempt to minimise costing bias and improve choice of instruments that will be used in our future RCT.

Aims:

- To develop and assess resource use data collection tools in support of the future economic evaluation within our definitive future RCT.
- To conduct micro-costing of both treatment pathways and to explore what are the determinants of variation in costs across settings and methods (macro- vs. micro-costing).
- To provide an economic rationale for the use of the most appropriate resource use identification, valuation and data collection tools.

Identifying what costs are to be included

A micro-costing approach to data identification and collection will be adopted. This approach will allow identification of resource use, meaning it will be focused and able to provide rich data about the resources used in relation to managing paediatric acute appendicitis in secondary care. Each stage of the data collection refers to event pathways for activity costing so that context and information is not lost in the final outcome of each activity. The process will include identification of services, how the service works and which components of costs are incurred upon delivery of each service. We will design and map processes involved in service delivery in order to identify all relevant resource use. Therefore, the details of the in-patient resource use will be collected through the design and implementation of Case Report Forms (CRFs) that will be informed from hospital records (from which medical history and previous and concurrent medication will be summarised), clinical and office charts, laboratory and pharmacy records, diaries, microfiches, radiographs, and correspondence. Detailed analysis of patients' hospital records will be undertaken for the first 10 patients recruited into CONTRACT across all three participating sites. This will enable an initial inclusive list of resource use items to be created and updated based on actual patient data using micro-costing principles. This work will inform the resource use data collection tool that will be used to collect data for the remainder of the recruited patients. Additionally, patient diary cards will be used to

Confidential: For Review Only

record resource use during the 14 days immediately following discharge from hospital. These will be used to collect data on use of antibiotics, pain medications and anti-inflammatory, or other relevant medications, as well as productivity loss and absence from school information. Finally, a modified version of the Client Service Receipt Inventory (CSRI) (16, 25) questionnaire will be used to collect other resource use data. The CSRI is a research instrument developed in the mid 1980s to collect information on service utilisation, income, accommodation and other cost-related variables. This will include health care appointments and additional family borne costs, as reported by parents of participants at 6-weeks following discharge and at 6-months.

Measuring and valuing resource use

Following identification of the patient's pathway and the services utilised, we will design a comprehensive list of resource use items that will be included in our resource inventory collection tool. This approach will not only form a comprehensive health profile of service utilisation that will form the outcome of this study, but will also lead to identification of the main cost drivers that need to be collected in our future definitive RCT. In this part of the study, we will use a mixed method approach for the valuation of the resources used. This valuation will utilise unit costs from both the PSSRU (Personal Social Services Research Unit) and the NHS Reference Costs data. Additionally, as part of our micro-costing approach we will collect and compare unit cost data from participating hospitals (14).

Data collection and analysis

After choosing the items of resource use to be included in this study, we will classify them in different components depending on the characteristics of the care pathway and service systems involved. Classifying resource use and costs implies focusing on variation at individual and aggregate level by trial arm. The economic sub-study at this stage will allow us to verify the relevance of this variation.

Both datasets of resource use and costs, at individual and aggregate level, will allow us to identify the main factors that influence the cost of the intervention, and will form the basis for considering the main cost drivers and methodology for inclusion in our definitive RCT. We will assess data quality and missing data identifying the most appropriate approach collecting economic data alongside randomised clinical

Confidential: For Review Only

studies. Descriptive statistics will be performed to summarise data and problems identified will be discussed and presented in a relevant publication. External validation will be achieved by comparing the outcomes from our bottom-up micro-costing to the NHS Reference Cost and the HRG tariff to identify the most appropriate costing method. We envisage that in case of significant variation in the costing methods we will be able to adopt both methods in our future cost-effectiveness analysis (CEA) in the form of sensitivity analysis. Given the importance of costs in any CEA, this proposed work will allow defining uncertainty around the CEA results and will provide an evidence base for future research.

PART II: PREFERENCE-BASED HRQoL INSTRUMENTS & THE QALY FRAMEWORK

The most commonly cited and used paediatric preference-based generic HRQoL instruments are the HUI (26), EQ-5D-Y (27) and CHU-9D (28). In the UK, there is a tendency towards the use of the EQ-5D-Y due to recommendation by NICE for adult population (EQ-5D-3L (29, 30)). The EQ-5D-Y comprises the same 3L as the adult version with improved wording for children despite not having a child specific value set. Euroqol states that *"Recent research has indicated that regular EQ-5D-3L value sets cannot be used for children and adolescents. The main reason is that health states are valued differently when described for an adult or a child."*
More recently a relatively new paediatric instrument, CHU-9D, has become more widely used in the UK. This is the only preference-based HRQoL measure specifically designed and developed with children using UK general population value sets. The HUI, although a paediatric instrument, was initially developed for patients with cancer and is not used as much in the UK. We believe this is due to two reasons: firstly, it relies on preference values obtained from the Canadian general population and not a UK population which might introduce some differences. Secondly, there is a cost attached to the use of HUI and this could be an issue for consideration when research studies need to operate under reasonably limited budget.

Principles of HRQoL assessment within CONTRACT

Confidential: For Review Only

To enable detection of any effect of our intervention on HRQoL we will collect data using two preference-based quality of life measures. The proposed measures are: (i) the EQ-5D-5L (31) which comprises the same 5 dimensions as the EQ-5D-3L and EQ-5D-Y but 5 levels of severity, which is considered to significantly increase reliability and sensitivity (discriminatory power) (31, 32) and (ii) the CHU-9D, the paediatric generic quality of life measure specifically designed for use in studies with children, which comprises 9 dimensions (28, 33-36). Both measures will be obtained from parent/carer proxy responses and children if 7-year or older.

Aims:

1. To compare two alternative preference-based generic HRQoL measures commonly used in paediatric studies.
2. To identify the most appropriate HRQoL instrument for economic evaluations alongside clinical studies for children with acute uncomplicated appendicitis in tertiary care settings.
3. To assess the variation and impact of time of data collection on utility values and the QALY framework when used in this population.

Data collection and analysis

We will collect both HRQoL measures at baseline, discharge, 2 weeks (to determine any short-term difference in QoL that may not be apparent at later follow-up), 6 weeks, 3 months, and 6 months, to define the most appropriate timing of assessment in relation to other health outcomes. Evidence from this work will support the decision for the most appropriate HRQoL instrument to be used, but also will provide valuable information adopting and reporting results in our future cost-utility analysis (CUA) in terms of cost per QALY gained. Any imbalances detected will inform sensitivity analyses and therefore, will enrich the results from the future definitive trial. We will also assess the appropriateness of using the QALY framework in this population, in terms of identifying aspects that are excluded from the conventional QALY framework, and aspects that the QALY framework could be sensitive in regards to timing of data collection.

CONCLUDING REMARKS

Costs of different interventions are an important part of any economic evaluation to determine whether a particular intervention is better placed, in terms of the outcomes

Confidential: For Review Only

it generates, in comparison to standard care (7, 8). The two most commonly used methods of collecting cost data are either “macro/top-down” or “micro/bottom-up” costing. The macro-costing uses the total budget to produce average costs per patient. This method is quicker but assumes that all patients have the same diagnosis, severity and treatment. Micro-costing measures resource use by individual patient, and therefore is considered more accurate detecting cost variability among patients. This method produces better quality costs but can be time-consuming and expensive (14). In this study we will assess two HRQoL measures and the implications of adopting the QALY framework in our future economic evaluation. Incorporating the outcomes from this economic sub-study into the feasibility stage of our RCT, and the micro-costing method we adopt in doing so, we believe it will enhance our results and their applicability for healthcare decision-making and for future economic evaluations.

Confidential: For Review Only

References:

1. Department of Health. NHS Reference Costs 2016-17. In: https://improvement.nhs.uk/resources/reference-costs/ LaM-J, editor. 2017.
2. Armstrong J, Merritt N, Jones S, Scott L, Bütter A. Non-operative management of early, acute appendicitis in children: Is it safe and effective? Journal of Pediatric Surgery. 2014;49(5):782-5.
3. Hartwich J, Luks FI, Watson-Smith D, Kurkchubasche AG, Muratore CS, Wills HE, et al. Nonoperative treatment of acute appendicitis in children: A feasibility study. J Pediatr Surg. 2016;51(1):111-6.
4. Minneci PC, Sulkowski JP, Nacion KM, Mahida JB, Cooper JN, Moss RL, et al. Feasibility of a nonoperative management strategy for uncomplicated acute appendicitis in children. J Am Coll Surg. 2014;219(2):272-9.
5. Svensson JF, Patkova B, Almstrom M, Naji H, Hall NJ, Eaton S, et al. Nonoperative treatment with antibiotics versus surgery for acute nonperforated appendicitis in children: a pilot randomized controlled trial. Ann Surg. 2015;261(1):67-71.
6. Hutchings N, Wood W, Reading I, Walker E, Blazeby JM, Van't Hoff W, et al. CONTRACT Study - CONservative TReatment of Appendicitis in Children (feasibility): study protocol for a randomised controlled Trial. Trials. 2018;19(1):153.
7. Drummond M MA. Economic evaluation in health care: merging theory with practice. 2nd ed. New York: Oxford University Press; 2004.
8. Drummond M, Sculpher M, Claxton K, Stoddart G, Torrance G. Methods for the economic evaluation of health care programmes. NY: Oxford University Press; 2015.
9. Luce BR, Elixhauser A. Estimating costs in the economic evaluation of medical technologies. Int J Technol Assess Health Care. 1990;6(1):57-75.
10. Riewpaiboon A, Malaroje S, Kongsawatt S. Effect of costing methods on unit cost of hospital medical services. Trop Med Int Health. 2007;12(4):554-63.
11. Smith MW, Barnett PG. Direct measurement of health care costs. Med Care Res Rev. 2003;60(3 Suppl):74s-91s.
12. Slothuus U. An Evaluation of Selected Literature on the Measurement of Costs in Health Economics. 2000.
13. Mogyorosy ZaS, P. The main methodological issues in costing health care services: A literature review. 2005.
14. Byford SM, D. Sefton, T. Because it's worth it: a practical guide to conducting economic evaluations in the social welfare field. London: York Publishing Services Ltd; 2003.
15. Heerey A, McGowan B, Ryan M, Barry M. Microcosting versus DRGs in the provision of cost estimates for use in pharmacoeconomic evaluation. Expert Rev Pharmacoecon Outcomes Res. 2002;2(1):29-33.
16. Beecham JaK, M. Costing psychiatric interventions. G. T, editor. London2001.
17. Knapp M, Beecham J. Reduced list costings: examination of an informed short cut in mental health research. Health Econ. 1993;2(4):313-22.
18. Garrison LP, Jr., Mansley EC, Abbott TA, 3rd, Bresnahan BW, Hay JW, Smeeding J. Good research practices for measuring drug costs in cost-effectiveness analyses: a societal perspective: the ISPOR Drug Cost Task Force report--Part II. Value Health. 2010;13(1):8-13.
19. Shearer J, McCrone P, Romeo R. Economic Evaluation of Mental Health Interventions: A Guide to Costing Approaches. Pharmacoeconomics. 2016;34(7):651-64.

Confidential: For Review Only

20. Oostenbrink JB, Koopmanschap MA, Rutten FF. Standardisation of costs: the Dutch Manual for Costing in economic evaluations. Pharmacoeconomics. 2002;20(7):443-54.
21. Barnett PG. An improved set of standards for finding cost for cost-effectiveness analysis. Med Care. 2009;47(7 Suppl 1):S82-8.
22. Husereau D, Drummond M, Petrou S, Carswell C, Moher D, Greenberg D, et al. Consolidated Health Economic Evaluation Reporting Standards (CHEERS) statement. BMJ : British Medical Journal. 2013;346.
23. Adam T, Koopmanschap MA, Evans DB. Cost-effectiveness analysis: can we reduce variability in costing methods? Int J Technol Assess Health Care. 2003;19(2):407-20.
24. Edwards RT, Charles JM, Lloyd-Williams H. Public health economics: a systematic review of guidance for the economic evaluation of public health interventions and discussion of key methodological issues. BMC Public Health. 2013;13:1001.
25. Beecham J. Collecting Information: the Client Service Receipt Interview. Mental Health Research. 1994;1,:6-8.
26. Horsman J, Furlong W, Feeny D, Torrance G. The Health Utilities Index (HUI(®)): concepts, measurement properties and applications. Health and Quality of Life Outcomes. 2003;1:54-.
27. Wille N, Badia X, Bonsel G, Burstrom K, Cavrini G, Devlin N, et al. Development of the EQ-5D-Y: a child-friendly version of the EQ-5D. Qual Life Res. 2010;19(6):875-86.
28. Stevens K. Assessing the performance of a new generic measure of health-related quality of life for children and refining it for use in health state valuation. Appl Health Econ Health Policy. 2011;9(3):157-69.
29. Dolan P. EuroQol--a new facility for the measurement of health-related quality of life. Health Policy. 1990;16(3):199-208.
30. Kind P, Dolan P, Gudex C, Williams A. Variations in population health status: results from a United Kingdom national questionnaire survey. Bmj. 1998;316(7133):736-41.
31. Herdman M, Gudex C, Lloyd A, Janssen M, Kind P, Parkin D, et al. Development and preliminary testing of the new five-level version of EQ-5D (EQ-5D-5L). Qual Life Res. 2011;20(10):1727-36.
32. Janssen MF, Pickard AS, Golicki D, Gudex C, Niewada M, Scalone L, et al. Measurement properties of the EQ-5D-5L compared to the EQ-5D-3L across eight patient groups: a multi-country study. Qual Life Res. 2013;22(7):1717-27.
33. Stevens K. Developing a descriptive system for a new preference-based measure of health-related quality of life for children. Qual Life Res. 2009;18(8):1105-13.
34. Stevens KJ, Freeman JV. An assessment of the psychometric performance of the Health Utilities Index 2 and 3 in children following discharge from a U.K. pediatric intensive care unit. Pediatr Crit Care Med. 2012;13(4):387-92.
35. Stevens K. Valuation of the Child Health Utility 9D Index. Pharmacoeconomics. 2012;30(8):729-47.
36. Stevens K, Ratcliffe J. Measuring and valuing health benefits for economic evaluation in adolescence: an assessment of the practicality and validity of the child health utility 9D in the Australian adolescent population. Value Health. 2012;15(8):1092-9.

Confidential: For Review Only

BMJ Paediatrics Open

Protocol of Health Economics and Quality of Life in a feasibility RCT of Paediatric Acute Appendicitis

Journal:	*BMJ Paediatrics Open*
Manuscript ID	bmjpo-2018-000347.R2
Article Type:	Protocol
Date Submitted by the Author:	31-Jul-2018
Complete List of Authors:	Chorozoglou, Maria; University of Southampton, Faculty of Medicine<br>Reading, Isabel ; University of Southampton , Primary Care and Population Sciences, Faculty of Medicine<br>Eaton, Simon; UCL Institute of Child Health,<br>Hutchings , Natalie; University of Southampton, Southampton Clinical Trials Unit, Faculty of Medicine<br>Hall, Nigel; University Surgery Unit
Keywords:	Health Economics, Paediatric Surgery, Costing

SCHOLARONE™
Manuscripts

Confidential: For Review Only

Protocol of Health Economics and Quality of Life in a feasibility RCT of Paediatric Acute Appendicitis

Authors:

Maria Chorozoglou (M.Chorozoglou@soton.ac.uk)[1]; Isabel Reading (i.c.reading@soton.ac.uk)[2]; Simon Eaton (s.eaton@ucl.ac.uk)[3]; Natalie Hutchings (n.j.hutchings@soton.ac.uk)[4]; and Nigel J Hall (n.j.hall@soton.ac.uk)[5,6]

Author for correspondence:

Maria Chorozoglou,
Senior Research Fellow - Health Economist
Southampton Health Technology Assessment Centre,
Faculty of Medicine, University of Southampton
The University of Southampton Science Park
Alpha House, Enterprise Road, Southampton SO17 1BJ, UK
E-mail: M.Chorozoglou@soton.ac.uk

Confidential: For Review Only

[1] Southampton Health Technology Assessment Centre (SHTAC), Faculty of Medicine, University of Southampton,
[2] Primary Care and Population Sciences, Faculty of Medicine, University of Southampton
[3] UCL Great Ormond Street Institute of Child Health
[4] Southampton Clinical Trials Unit, Faculty of Medicine, University of Southampton
[5] Department of Paediatric Surgery and Urology, Southampton Children's Hospital, University Hospital Southampton NHS Foundation Trust, UK
[6] University Surgery Unit, Faculty of Medicine, University of Southampton

What is known about the subject – followed by a maximum of 3 brief statements (no more than 25 words per statement)

1. Acute appendicitis is one of the most common acute surgical emergencies in children and there is great current interest in the role of non-operative treatment.
2. The economic impact of different treatment options on the health system (e.g. NHS) if adopted remains largely unknown.
3. The quality of any economic evaluation depends on the quality of the measurement of costs and outcomes, despite this very few HE protocols are published.

What this study hopes to add – followed by a maximum of 3 brief statements (no more than 25 words per statement)

1. This study proposes a comprehensive methodological approach that could be adopted in an early stage of assessment of a medical technology.
2. To identify the most appropriate HRQoL instrument for children with acute uncomplicated appendicitis in tertiary care settings.
3. Adds to evidence base in paediatric surgical research in terms of methodology of economic studies.

- **Authors' contributions**: state how each author was involved in writing the protocol

The lead health economist (MC) designed the Health Economic Analysis Plan and prepared this manuscript; the lead statistician (IR) contributed to the design of the study; the principal investigator (NH) is involved in all aspects of the study; All co-authors contributed to the preparation and approval of this manuscript.

- **Funding statement**: preferably worded in one of two ways:

The authors acknowledge funding received from the UK National Institute for Health Research (NIHR) Health Technology Assessment (HTA) Board (14/192/90)

- **Competing interests statement**

No competing interest has been declared from any of the authors.

Confidential: For Review Only

ABSTRACT

Background: Acute appendicitis is one of the most common acute surgical emergencies in children and accounts for an annual cost of approximately £50 million to the NHS. Investigating alternative treatment options offers not only the best prospect of enhancing the quality of care for patients but also potential opportunities for cost savings through better allocative efficiency. A feasibility Randomised Controlled Trial (RCT) comparing a non-operative treatment pathway with appendicectomy for children with acute uncomplicated appendicitis is underway (CONTRACT feasibility RCT).

Aims: The prime objective of this economic sub-study conducted alongside the CONTRACT feasibility RCT is to better understand and assess: (i) cost data collection tools and cost drivers by identifying patients' pathways and (ii) patient Quality of Life (QoL) by assessing alternative paediatric Health Related Quality of Life (HRQoL) instruments. Outcomes from this study will inform a future efficacy RCT assessing the effectiveness and cost-effectiveness of non-operative treatment pathway for the treatment of acute uncomplicated appendicitis in children.

Methods: The economic sub-study will use individual-level data and will be conducted from the health system perspective over the study's 6-month follow-up period. Micro-costing will include health resource and service use, while potential benefits acquired will be measured using the HRQoL measures, CHU-9D and EQ-5D-5L. We will assess the appropriateness of using the cost per QALY framework in the future RCT, as well as testing and identifying the most suitable HRQoL instrument.

Conclusions: The outcomes of the investigational economic sub-study will be used to inform the design of our future definitive RCT. However, the result from this economic study will also provide a detailed description and account of the issues inherent in paediatric Economic Evaluations Alongside Clinical Trials (EEACT) with an emphasis on costing methods of interventions taking place in secondary care settings.

Word count:
Abstract 292
Main manuscript 2587

Confidential: For Review Only

INTRODUCTION

Background

Acute appendicitis is one of the most common acute surgical emergencies in children. According to the National Schedule of Reference Costs/HRG (Healthcare Resource Groups) data, almost fourteen thousand operations are performed every year in the UK (<18y). Appendicectomy procedure for a patient under 18 years old, costs on average from £3,072 to £5,992 and accounts for an annual total cost of approximately £50 million to the NHS (1).

For many years, appendicectomy has been considered the standard treatment for appendicitis, both in adults and in children. However, there is great current interest in the role of non-operative treatment (with antibiotics alone) of adults and children with acute appendicitis. Alongside evaluations of the clinical effectiveness of this alternative treatment option, it is equally important to explore the economic implications. A small number of studies have reported (mainly clinical) outcomes of non-operative treatment of acute appendicitis in children, but very little is published on how this translates into economic outcomes (2-5). Therefore, the economic impact of different treatment options on the health system (e.g. NHS) if adopted remains largely unknown. We plan to conduct a definitive RCT comparing these alternative treatment options. Since recruitment to such a RCT will be challenging, we are first conducting a feasibility RCT in the UK (6). Alongside this feasibility RCT, we will conduct a health economic feasibility sub-study to inform a future cost-effectiveness and cost-utility analysis within our definitive RCT. Herein we describe the protocol for this health economic sub-study. We aim to address the following research questions: what are the cost implications of treating childhood appendicitis non-operatively as compared to surgery; how the costs of both treatment options compare to widely used NHS Reference Costs; and what could be the implications of differing cost methods in assessing the cost-effectiveness of appendicitis. The study will also assess two preference-based QoL questionnaires widely used in paediatric research, using as reference case, clinical outcomes identified through the feasibility study.

Study design, participants, interventions, outcomes

Confidential: For Review Only

The CONservative TReatment of Appendicitis in Children – randomised controlled Trial (CONTRACT) study is a feasibility RCT that aims to explore whether it is feasible and acceptable to conduct a multi-centre randomised controlled trial testing the effectiveness and cost-effectiveness of a non-operative treatment pathway for the treatment of acute uncomplicated appendicitis in children. The study is being conducted in three specialist NHS Paediatric Units in England and participants are children (age 4-15 years) with a clinical diagnosis of acute uncomplicated appendicitis. It has been estimated that 52-65 participants in the feasibility RCT will be adequate to test treatment pathway procedures. The health technology under assessment involves treatment with antibiotics (intravenous followed by oral) and regular clinical review to determine disease resolution without appendicectomy, or appendicectomy in those whom the disease worsens or fails to resolve. The broad inclusion criteria reflect current clinical practice and enable the generalisability of our results for routine in-patient care. The principal outcome of the feasibility study will be recruitment rate. A full protocol of the CONTRACT feasibility RCT is published elsewhere (6). The study, including the economic sub-study, has been approved by the South Central – Hampshire A Research Ethics Committee (16/SC/0596). The feasibility RCT was registered on 8th February 2017 (http://www.isrctn.com/ISRCTN15830435).

Scope of the economic sub-study protocol

The economic sub-study will provide evidence and guidance for determining data collection tools measuring cost and benefit outcomes for our future RCT assessing the cost-effectiveness of the non-operative treatment of appendicitis. Data management will be performed by the Southampton Clinical Trials Unit (SCTU) and anonymised data will be delivered for the health economic analysis. This protocol describes methods for incorporating economic evidence into an early stage of the study and has been conceptually divided into two parts: (i) measuring resource utilisation and conducting micro-costing, and (ii) measuring QoL and assessment of HRQoL instruments.

METHODS AND ANALYSIS

PART I: RESOURCE UTILISATION & COSTS

Confidential: For Review Only

Costs are an important part of any economic evaluation but is a term that has different meaning across different disciplines. In Health Economics, costs are related to opportunity costs and the question of interest is the choice between two alternatives, in other words there are always forgone opportunities when choosing to invest in a new medical technology or health service rather than in a current treatment (7, 8).

Principles of costing within CONTRACT

The quality of the economic evaluation depends on the quality of the measurement of costs and outcomes (8-10). There are two main approaches used to measure health care costs: the "macro-costing/top-down" and the "micro-costing/bottom-up" approach (8, 11-14). The gross or macro-costing method is commonly used in cost analysis providing an overview of the effect of costs, but it has been argued that it is not appropriate in many cases for economic evaluation because it provides limited accuracy and detail (8, 14). In the UK, it is common practice to use the NHS Reference Costs/HRGs which provides National tariffs as the unit costs for different services and procedures. These unit costs are calculated using the mean costs among patients and hospitals. However, these national tariffs might not represent good estimates of real costs especially for new interventions and some reports show cost estimate discrepancies between macro-costing and micro-costing, ranging between 9% and 66% (15). Advocates of the macro-costing approach highlight the advantages of this method, namely generalisability, easy to use and less time consuming. Micro-costing reflects the individual patient costs by identifying and collecting the actual individual resources used and estimating the economic costs of resources utilised. However, despite the micro-costing approach being regarded as more accurate and transparent, it is considered time consuming and not easily applied in some settings (14). A general rule recommended by Beecham and Knapp (16) is that the broader and most accurate approach is collecting individual data through a micro-costing method or adopting a "reduced list costing" (17) which implies the selection of a limited number of services considered to be of most significance.

Detailed recommended methodology on costing is still lacking but several guidelines have emerged (13, 18-21) such as a series of task force reports on methodological issues in costing methods by the International Society for Pharmacoeconomics and

Confidential: For Review Only

Outcomes Research (20, 22). However, guidelines still vary in terms of recommended methods and variations of costing methods affect the validity and comparability of economic evaluation results (23, 24). Our approach in this economic study is to compare the two methods in an attempt to minimise costing bias and improve choice of instruments that will be used in our future RCT.

Aims:

- To develop and assess resource use data collection tools in support of the future economic evaluation within our definitive future RCT.
- To conduct micro-costing of both treatment pathways and to explore what are the determinants of variation in costs across settings and methods (macro- vs. micro-costing).
- To provide an economic rationale for the use of the most appropriate resource use identification, valuation and data collection tools.

Identifying what costs are to be included

A micro-costing approach to data identification and collection will be adopted. This approach will allow identification of resource use, meaning it will be focused and able to provide rich data about the resources used in relation to managing paediatric acute appendicitis in secondary care. Each stage of the data collection refers to event pathways for activity costing so that context and information is not lost in the final outcome of each activity. The process will include identification of services, how the service works and which components of costs are incurred upon delivery of each service. We will design and map processes involved in service delivery in order to identify all relevant resource use. Therefore, the details of the in-patient resource use will be collected through the design and implementation of Case Report Forms (CRFs) that will be informed from hospital records (from which medical history and previous and concurrent medication will be summarised), clinical and office charts, laboratory and pharmacy records, diaries, microfiches, radiographs, and correspondence. Detailed analysis of patients' hospital records will be undertaken for the first 10 patients recruited into CONTRACT across all three participating sites. This will enable an initial inclusive list of resource use items to be created and updated based on actual patient data using micro-costing principles. This work will inform the resource use data collection tool that will be used to collect data for the remainder of the recruited patients. Additionally, patient diary cards will be used to

Confidential: For Review Only

record resource use during the 14 days immediately following discharge from hospital. These will be used to collect data on use of antibiotics, pain medications and anti-inflammatory, or other relevant medications, as well as productivity loss and absence from school information. Finally, a modified version of the Client Service Receipt Inventory (CSRI) (16, 25) questionnaire will be used to collect other resource use data. The CSRI is a research instrument developed in the mid 1980s to collect information on service utilisation, income, accommodation and other cost-related variables. This will include health care appointments and additional family borne costs, as reported by parents of participants at 6-weeks following discharge and at 6-months.

Measuring and valuing resource use

Following identification of the patient's pathway and the services utilised, we will design a comprehensive list of resource use items that will be included in our resource inventory collection tool. This approach will not only form a comprehensive health profile of service utilisation that will form the outcome of this study, but will also lead to identification of the main cost drivers that need to be collected in our future definitive RCT. In this part of the study, we will use a mixed method approach for the valuation of the resources used. This valuation will utilise unit costs from both the PSSRU (Personal Social Services Research Unit) and the NHS Reference Costs data. Additionally, as part of our micro-costing approach we will collect and compare unit cost data from participating hospitals (14).

Data collection and analysis

After choosing the items of resource use to be included in this study, we will classify them in different components depending on the characteristics of the care pathway and service systems involved. Classifying resource use and costs implies focusing on variation at individual and aggregate level by trial arm. The economic sub-study at this stage will allow us to verify the relevance of this variation.

Both datasets of resource use and costs, at individual and aggregate level, will allow us to identify the main factors that influence the cost of the intervention, and will form the basis for considering the main cost drivers and methodology for inclusion in our definitive RCT. We will assess data quality and missing data identifying the most appropriate approach collecting economic data alongside randomised clinical

Confidential: For Review Only

studies. Descriptive statistics will be performed to summarise data and problems identified will be discussed and presented in a relevant publication. External validation will be achieved by comparing the outcomes from our bottom-up micro-costing to the NHS Reference Cost and the HRG tariff to identify the most appropriate costing method. We envisage that in case of significant variation in the costing methods we will be able to adopt both methods in our future cost-effectiveness analysis (CEA) in the form of sensitivity analysis. Given the importance of costs in any CEA, this proposed work will allow defining uncertainty around the CEA results and will provide an evidence base for future research.

PART II: PREFERENCE-BASED HRQoL INSTRUMENTS & THE QALY FRAMEWORK

The most commonly cited and used paediatric preference-based generic HRQoL instruments are the HUI (26), EQ-5D-Y (27) and CHU-9D (28). In the UK, there is a tendency towards the use of the EQ-5D-Y due to recommendation by NICE for adult population (EQ-5D-3L (29, 30)). The EQ-5D-Y comprises the same 3L as the adult version with improved wording for children despite not having a child specific value set. Euroqol states that *“Recent research has indicated that regular EQ-5D-3L value sets cannot be used for children and adolescents. The main reason is that health states are valued differently when described for an adult or a child.”*

More recently a relatively new paediatric instrument, CHU-9D, has become more widely used in the UK. This is the only preference-based HRQoL measure specifically designed and developed with children using UK general population value sets. The HUI, although a paediatric instrument, was initially developed for patients with cancer and is not used as much in the UK. We believe this is due to two reasons: firstly, it relies on preference values obtained from the Canadian general population and not a UK population which might introduce some differences. Secondly, there is a cost attached to the use of HUI and this could be an issue for consideration when research studies need to operate under reasonably limited budget.

Principles of HRQoL assessment within CONTRACT

Confidential: For Review Only

To enable detection of any effect of our intervention on HRQoL we will collect data using two preference-based quality of life measures. The proposed measures are: (i) the EQ-5D-5L (31) which comprises the same 5 dimensions as the EQ-5D-3L and EQ-5D-Y but 5 levels of severity, which is considered to significantly increase reliability and sensitivity (discriminatory power) (31, 32) and (ii) the CHU-9D, the paediatric generic quality of life measure specifically designed for use in studies with children, which comprises 9 dimensions (28, 33-36). Both measures will be obtained from parent/carer proxy responses and children if 7-year or older.

Aims:

1. To compare two alternative preference-based generic HRQoL measures commonly used in paediatric studies.
2. To identify the most appropriate HRQoL instrument for economic evaluations alongside clinical studies for children with acute uncomplicated appendicitis in tertiary care settings.
3. To assess the variation and impact of time of data collection on utility values and the QALY framework when used in this population.

Data collection and analysis

We will collect both HRQoL measures at baseline, discharge, 2 weeks (to determine any short-term difference in QoL that may not be apparent at later follow-up), 6 weeks, 3 months, and 6 months, to define the most appropriate timing of assessment in relation to other health outcomes. Evidence from this work will support the decision for the most appropriate HRQoL instrument to be used, but also will provide valuable information adopting and reporting results in our future cost-utility analysis (CUA) in terms of cost per QALY gained. Any imbalances detected will inform sensitivity analyses and therefore, will enrich the results from the future definitive trial. We will also assess the appropriateness of using the QALY framework in this population, in terms of identifying aspects that are excluded from the conventional QALY framework, and aspects that the QALY framework could be sensitive in regards to timing of data collection.

CONCLUDING REMARKS

Costs of different interventions are an important part of any economic evaluation to determine whether a particular intervention is better placed, in terms of the outcomes

Confidential: For Review Only

it generates, in comparison to standard care (7, 8). The two most commonly used methods of collecting cost data are either “macro/top-down” or “micro/bottom-up” costing. The macro-costing uses the total budget to produce average costs per patient. This method is quicker but assumes that all patients have the same diagnosis, severity and treatment. Micro-costing measures resource use by individual patient, and therefore is considered more accurate detecting cost variability among patients. This method produces better quality costs but can be time-consuming and expensive (14). In this study we will assess two HRQoL measures and the implications of adopting the QALY framework in our future economic evaluation. Incorporating the outcomes from this economic sub-study into the feasibility stage of our RCT, and the micro-costing method we adopt in doing so, we believe it will enhance our results and their applicability for healthcare decision-making and for future economic evaluations.

Confidential: For Review Only

References:

1. Department of Health. NHS Reference Costs 2016-17. In: https://improvement.nhs.uk/resources/reference-costs/ LaM-J, editor. 2017.
2. Armstrong J, Merritt N, Jones S, Scott L, Bütter A. Non-operative management of early, acute appendicitis in children: Is it safe and effective? Journal of Pediatric Surgery. 2014;49(5):782-5.
3. Hartwich J, Luks FI, Watson-Smith D, Kurkchubasche AG, Muratore CS, Wills HE, et al. Nonoperative treatment of acute appendicitis in children: A feasibility study. J Pediatr Surg. 2016;51(1):111-6.
4. Minneci PC, Sulkowski JP, Nacion KM, Mahida JB, Cooper JN, Moss RL, et al. Feasibility of a nonoperative management strategy for uncomplicated acute appendicitis in children. J Am Coll Surg. 2014;219(2):272-9.
5. Svensson JF, Patkova B, Almstrom M, Naji H, Hall NJ, Eaton S, et al. Nonoperative treatment with antibiotics versus surgery for acute nonperforated appendicitis in children: a pilot randomized controlled trial. Ann Surg. 2015;261(1):67-71.
6. Hutchings N, Wood W, Reading I, Walker E, Blazeby JM, Van't Hoff W, et al. CONTRACT Study - CONservative TReatment of Appendicitis in Children (feasibility): study protocol for a randomised controlled Trial. Trials. 2018;19(1):153.
7. Drummond M MA. Economic evaluation in health care: merging theory with practice. 2nd ed. New York: Oxford University Press; 2004.
8. Drummond M, Sculpher M, Claxton K, Stoddart G, Torrance G. Methods for the economic evaluation of health care programmes. NY: Oxford University Press; 2015.
9. Luce BR, Elixhauser A. Estimating costs in the economic evaluation of medical technologies. Int J Technol Assess Health Care. 1990;6(1):57-75.
10. Riewpaiboon A, Malaroje S, Kongsawatt S. Effect of costing methods on unit cost of hospital medical services. Trop Med Int Health. 2007;12(4):554-63.
11. Smith MW, Barnett PG. Direct measurement of health care costs. Med Care Res Rev. 2003;60(3 Suppl):74s-91s.
12. Slothuus U. An Evaluation of Selected Literature on the Measurement of Costs in Health Economics. 2000.
13. Mogyorosy ZaS, P. The main methodological issues in costing health care services: A literature review. 2005.
14. Byford SM, D. Sefton, T. Because it's worth it: a practical guide to conducting economic evaluations in the social welfare field. London: York Publishing Services Ltd; 2003.
15. Heerey A, McGowan B, Ryan M, Barry M. Microcosting versus DRGs in the provision of cost estimates for use in pharmacoeconomic evaluation. Expert Rev Pharmacoecon Outcomes Res. 2002;2(1):29-33.
16. Beecham JaK, M. Costing psychiatric interventions. G. T, editor. London2001.
17. Knapp M, Beecham J. Reduced list costings: examination of an informed short cut in mental health research. Health Econ. 1993;2(4):313-22.
18. Garrison LP, Jr., Mansley EC, Abbott TA, 3rd, Bresnahan BW, Hay JW, Smeeding J. Good research practices for measuring drug costs in cost-effectiveness analyses: a societal perspective: the ISPOR Drug Cost Task Force report--Part II. Value Health. 2010;13(1):8-13.
19. Shearer J, McCrone P, Romeo R. Economic Evaluation of Mental Health Interventions: A Guide to Costing Approaches. Pharmacoeconomics. 2016;34(7):651-64.

Confidential: For Review Only

20. Oostenbrink JB, Koopmanschap MA, Rutten FF. Standardisation of costs: the Dutch Manual for Costing in economic evaluations. Pharmacoeconomics. 2002;20(7):443-54.
21. Barnett PG. An improved set of standards for finding cost for cost-effectiveness analysis. Med Care. 2009;47(7 Suppl 1):S82-8.
22. Husereau D, Drummond M, Petrou S, Carswell C, Moher D, Greenberg D, et al. Consolidated Health Economic Evaluation Reporting Standards (CHEERS) statement. BMJ : British Medical Journal. 2013;346.
23. Adam T, Koopmanschap MA, Evans DB. Cost-effectiveness analysis: can we reduce variability in costing methods? Int J Technol Assess Health Care. 2003;19(2):407-20.
24. Edwards RT, Charles JM, Lloyd-Williams H. Public health economics: a systematic review of guidance for the economic evaluation of public health interventions and discussion of key methodological issues. BMC Public Health. 2013;13:1001.
25. Beecham J. Collecting Information: the Client Service Receipt Interview. Mental Health Research. 1994;1,:6-8.
26. Horsman J, Furlong W, Feeny D, Torrance G. The Health Utilities Index (HUI(®)): concepts, measurement properties and applications. Health and Quality of Life Outcomes. 2003;1:54-.
27. Wille N, Badia X, Bonsel G, Burstrom K, Cavrini G, Devlin N, et al. Development of the EQ-5D-Y: a child-friendly version of the EQ-5D. Qual Life Res. 2010;19(6):875-86.
28. Stevens K. Assessing the performance of a new generic measure of health-related quality of life for children and refining it for use in health state valuation. Appl Health Econ Health Policy. 2011;9(3):157-69.
29. Dolan P. EuroQol--a new facility for the measurement of health-related quality of life. Health Policy. 1990;16(3):199-208.
30. Kind P, Dolan P, Gudex C, Williams A. Variations in population health status: results from a United Kingdom national questionnaire survey. Bmj. 1998;316(7133):736-41.
31. Herdman M, Gudex C, Lloyd A, Janssen M, Kind P, Parkin D, et al. Development and preliminary testing of the new five-level version of EQ-5D (EQ-5D-5L). Qual Life Res. 2011;20(10):1727-36.
32. Janssen MF, Pickard AS, Golicki D, Gudex C, Niewada M, Scalone L, et al. Measurement properties of the EQ-5D-5L compared to the EQ-5D-3L across eight patient groups: a multi-country study. Qual Life Res. 2013;22(7):1717-27.
33. Stevens K. Developing a descriptive system for a new preference-based measure of health-related quality of life for children. Qual Life Res. 2009;18(8):1105-13.
34. Stevens KJ, Freeman JV. An assessment of the psychometric performance of the Health Utilities Index 2 and 3 in children following discharge from a U.K. pediatric intensive care unit. Pediatr Crit Care Med. 2012;13(4):387-92.
35. Stevens K. Valuation of the Child Health Utility 9D Index. Pharmacoeconomics. 2012;30(8):729-47.
36. Stevens K, Ratcliffe J. Measuring and valuing health benefits for economic evaluation in adolescence: an assessment of the practicality and validity of the child health utility 9D in the Australian adolescent population. Value Health. 2012;15(8):1092-9.

Confidential: For Review Only
