# Supplementary material for: Health economics and quality of life in a feasibility RCT of paediatric acute appendicitis: a protocol study
Source: BMJ Paediatr Open. 2018 Sep 21;2(1):e000347. doi: 10.1136/bmjpo-2018-000347 (PMC6157565; doi:10.1136/bmjpo-2018-000347)
Supplement: Reviewer comments [file bmjpo-2018-000347.reviewer_comments.pdf]

PEER REVIEW HISTORY

BMJ Paediatrics Open publishes all reviews undertaken for accepted manuscripts. Reviewers are asked to complete a checklist review form and are provided with free text boxes to elaborate on their assessment. These free text comments are reproduced below.

ARTICLE DETAILS

**TITLE (PROVISIONAL)**	Protocol of Health Economics and Quality of Life in a feasibility RCT of Paediatric Acute Appendicitis
**AUTHORS**	Chorozoglou, Maria; Reading, Isabel; Eaton, Simon; Hutchings, Natalie; Hall, Nigel

This article was not externally reviewed at BMJ Paediatrics Open.
